# The Development of a Fatigue Failure Prediction Model for Bitumen Based on a Novel Accelerated Cyclic Shear Test

**DOI:** 10.3390/ma18163729

**Published:** 2025-08-08

**Authors:** Yankai Wen, Lin Wang

**Affiliations:** Department of Civil and Environmental Engineering, Washington State University, Pullman, WA 99163, USA; yankai.wen@wsu.edu

**Keywords:** fatigue failure, failure mechanism, fatigue life, load ratio, sigmoidal model

## Abstract

Fatigue failure of bitumen significantly influences the durability and service life of asphalt pavement. Current fatigue tests have drawbacks such as long durations, unrealistic traffic loading simulations, and difficulties of identifying failure mechanisms. Similarly, existing prediction models are often overly complex and inaccurate. To solve these drawbacks, in this study, a novel accelerated cyclic shear test in stress-controlled mode using a dynamic shear rheometer was introduced to evaluate the fatigue performance and reveal the fatigue failure mechanism of bitumen. The sigmoidal function was applied to develop a simplified fatigue failure prediction model for bitumen through stress and temperature shifts. The results demonstrate that bitumen’s response under the newly proposed loading method aligns consistently with behaviour characteristic of a plasticity-controlled failure mechanism. The variable parameter load ratio significantly influenced the bitumen’s time-to-failure, which increased as the load ratio decreased. Bitumen exhibited the longest time-to-failure when the load ratio (minimum stress/maximum stress) was 0.1. The developed model effectively predicted the time-to-failure of bitumen across different load ratios and under various temperature and stress conditions.

## 1. Introduction

Fatigue failure represents a critical concern for asphalt pavement throughout its service life [1,2]. Repeated traffic loading and varying climatic conditions contribute to the progressive development of permanent deformation and microdamage, ultimately resulting in fatigue damage and eventual failure. Fatigue failure typically occurs either at the bitumen–aggregate interface, leading to adhesive failure, or within the bitumen itself, resulting in cohesive failure [3,4]. Bitumen also interacts with mineral filler to form asphalt mastic, whose fatigue resistance is significantly influenced by both the origin and the quantity of the filler [5]. As the binder is between aggregate particles, bitumen is critical to the fatigue resistance of asphalt mixtures. Therefore, evaluating its performance accurately prior to field application is essential.

The strategic highway research program (SHRP) introduced the fatigue factor (G*sinδ), measured through a dynamic shear rheometer (DSR) oscillation test, as an early rheological parameter for quantifying the fatigue resistance of bitumen [6]. However, its validity has been questioned, as G*sinδ is a stiffness-based parameter measured under simplified conditions that fails to accurately reflect the complex loading and temperature variations experienced by bitumen in real-world pavement [7,8,9,10]. The Glover–Rowe parameter ($G^{\prime 2}/G^{\prime \prime }$) is another fatigue index that was proposed to assess the fatigue resistance of bitumen [11,12]. Ilyin and Yadykova [13] applied equation $\frac{G^{\prime 2}}{G^{\prime \prime }}<1.8\cdot 10^{5}Pa$ at an angular frequency of 0.005 rad/s and a temperature of 15 °C to investigate the fatigue resistance of bitumen. The time sweep (TS) test is a well-established method for assessing the fatigue properties of bitumen. Numerous researchers have developed viscoelastic indices and fatigue failure criteria based on TS experimental studies to improve our understanding of bitumen fatigue behaviour [10,14,15,16,17]. However, some fatigue criteria depend on the applied load-controlled mode (stress-controlled or strain-controlled), such as the plateau value (PV) energy parameter [18,19]. Additionally, the TS test is time-consuming due to its slow damage growth. The TS test typically applies load amplitudes within the linear viscoelastic (LVE) range of bitumen. However, in real-world conditions, bitumen often experiences loading beyond this range, leading to nonlinear viscoelastic and viscoplastic responses [6,20]. Chen et al. [21] reported that the damage resistance of bitumen under large-amplitude fatigue loading exhibits a stronger correlation with the fatigue performance of asphalt mixtures, as compared to small-amplitude fatigue loadings. Concurrently, extensive research has been conducted to develop novel fatigue tests for bitumen. Bahia and colleagues developed the linear amplitude sweep (LAS) test [22], which subjects bitumen to oscillatory shear strain with progressively increasing amplitudes until failure occurs. When combined with viscoelastic continuum damage (VECD) analysis, the LAS test has been effectively utilized for fatigue evaluation [23,24,25]. Zhang and Oeser [26] observed that the damage mechanism of bitumen varies with the applied load amplitude, which means that different load amplitudes potentially reflect distinct fatigue failure mechanisms. From a rheological perspective, bitumen exhibits increased flow behaviour under high shear stresses, indicating that plastic flow damage predominates the deterioration process [26,27].

Most current research primarily investigates the damage and failure of bitumen based on its viscoelastic response, while relatively few studies have investigated the significant influence of plasticity on the deformation and failure mechanism of bitumen. Unlike elastic deformation, which is reversible upon unloading, plastic deformation leads to permanent strain accumulation. For instance, in DSR tests at high temperatures or stress/strain amplitudes, bitumen may exhibit irreversible deformation, which cannot be characterized by purely viscoelastic models. Studies [28,29,30] on polymers have consistently emphasized the crucial role of plastic components in materials’ fatigue failure by defining a plasticity-controlled failure mechanism. Some studies [28,31,32] have demonstrated that plasticity controlling can assess the failure mechanisms of polymers in both cyclic and creep tests. Bitumen exhibits behaviour similar to these polymers, characterized by increased molecular mobility and progressive deformation under specific loading conditions, ultimately leading to a constant plastic flow [11,33,34]. Therefore, it is essential to improve existing methods by considering plasticity. This will enhance the understanding of the plastic flow and failure of bitumen from a plasticity perspective and support the development of more accurate prediction models.

This study aims to bridge existing gaps and enhance the evaluation of the fatigue performance of bitumen under diverse loading paths. A novel stress-controlled accelerated cyclic shear test was developed by regulating the load ratio *R* (*R* = minimum stress/maximum stress) through varying oscillation amplitudes while maintaining the steady stress, which can effectively combine features of both creep and cyclic loading tests. A new index, time-to-failure (*t_f_*), was defined as the key parameter of fatigue behaviour. The fatigue failure mechanism of bitumen under the novel loading mode was investigated. A sigmoidal function was utilized to develop a fatigue failure model capable of predicting fatigue behaviour across various *R* values, stress levels, and temperatures. The model’s accuracy was validated by comparing the experimental results with the predicted results generated by the developed fatigue model.

## 2. Methodology

### 2.1. Accelerated Cyclic Shear Test

**Figure 1:**
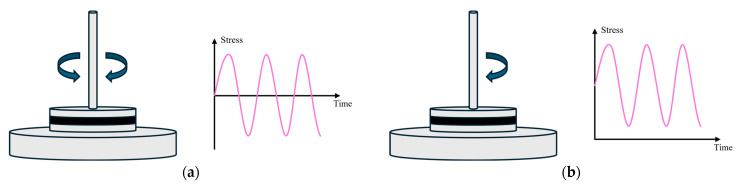
Schematic illustration of (**a**) bidirectional sinusoidal oscillating loading and (**b**) the new unidirectional sinusoidal cyclic loading applied by the DSR.

**Figure 2:**
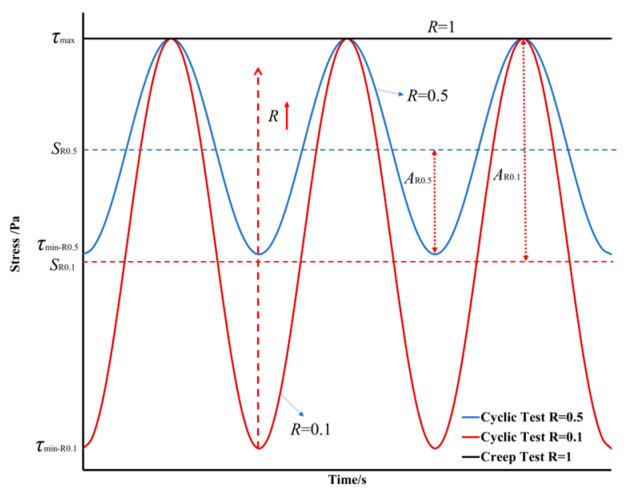
Schematic illustration of the accelerated cyclic shear loading.

The cyclic loading test is a commonly employed method for assessing the fatigue performance of bitumen. However, under the bidirectional sinusoidal oscillating loading that is commonly applied by the DSR, as shown in Figure 1a, the resulting plastic deformation is partially offset, introducing challenges in accurately estimating the fatigue life of bitumen under realistic traffic loading. To address this drawback, this study introduces an accelerated cyclic shear test by incorporating the static loading and cyclic sinusoidal loading to achieve new unidirectional sinusoidal cyclic loading, as shown in Figure 1b. This loading can be characterized by the maximum stress (*τ*_max_), minimum stress (*τ*_min_), steady stress (*S*), and load amplitude (*A*). These parameters are expressed in terms of load ratio (*R*), as defined in Equation (1). This loading mode establishes a correlation between the creep test and cyclic test through *R*, as the cyclic test with *R* = 1 is the creep test. The details of the applied cyclic loads are illustrated in Figure 2. It can be found that a load ratio of *R* = 1 corresponds to creep tests, while values of *R* < 1 indicate cyclic tests. Furthermore, as the *R* decreases, the load amplitude increases.

(1) $$R=\frac{\tau_{min}}{\tau_{max}}=\frac{\tau_{min}}{2A+\tau_{min}}$$

where *R* is the load ratio, *τ*_min_ is the minimum value of the cyclic load, *τ*_max_ is the maximum value of the cyclic load, and *A* is the cyclic load amplitude.

**Figure 3:**
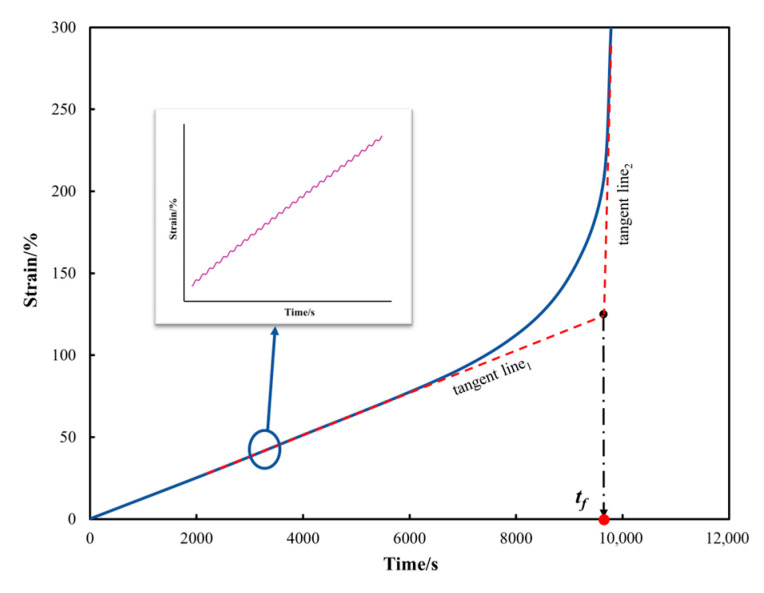
A schematic illustration of the geometric method used to determine the *t_f_*.

In this study, a new time-to-failure (*t_f_*) index is defined using a straightforward geometrical method to represent the fatigue failure of bitumen, which overcomes the complex calculations associated with existing failure indices. The *t_f_* is defined as the time corresponding to the intersection point of the tangents of the second and third stages of the accumulated strain curve. Figure 3 is the schematic illustration of the geometric method used to determine the *t_f_* under the novel accelerated cyclic shear test.

### 2.2. Materials and Experiments

The PG58-28 and PG64-22 bitumen used in this study were randomly sourced from Washington and Idaho States in the United States, respectively. The properties of the bitumen are listed in Table 1. Given that the accelerated cyclic shear test introduced in this study represents a novel experimental method, only the two original (unmodified and unaged) bitumen types were evaluated in this study. Modified and aged bitumen were not considered in this study but will be addressed in future research.

All tests in this study were performed under a stress-controlled model by a Malvern Gemini 150 dynamic shear rheometer (DSR) with an 8 mm diameter plate and 2 mm gap parallel plates setup according to AASHTO T 315 [35]. The selection of test temperatures in this study considered the high-temperature limitation of the 8 mm parallel plate setup and the potential risk of debonding at the interface between the parallel plates and specimen. Therefore, the selected temperatures were in the medium temperature range of 20–45 °C. To ensure accurate deformation data, all tests were conducted in duplicate, and the results reported in this paper represent the average values of the two measurements. Creep tests (*R* = 1) and cyclic tests (*R* < 1) were performed over a wide range of stresses that were carefully chosen for each bitumen and temperature to obtain failure within a time scale ranging from 10^2^ s to 10^5^ s. The *R* was changed according to the test requirements for every cyclic test, which was set to increase gradually from 0.1 to 1 (*R* = 0.1, 0.3, 0.5, 0.7, 1). In all cyclic tests, the loading frequency was maintained at a constant 10 Hz. The test matrixes are listed in Table 2. The *t_f_* was utilized as an evaluation index for assessing the failure of bitumen. For the test results, data obtained at 20 °C, 35 °C, and 45 °C were used for the analysis and development of the fatigue failure model, while the experimental data at 30 °C and 40 °C were reserved for model validation.

## 3. Results and Analysis

### 3.1. Time-to-Failure

**Figure 4:**
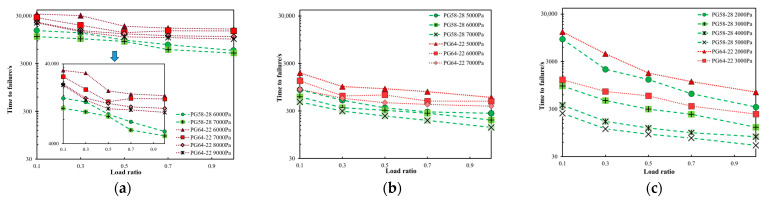
Time-to-failure of PG58-28 and PG64-22 bitumen under different load ratios at (**a**) 20 °C (**b**) 35 °C (**c**) 45 °C.

Figure 4 illustrates the relationship between the *R* and the *t_f_* for PG58-28 and PG64-22 bitumen under various stresses and temperatures. Under the same maximum stress, the *t_f_* decreases as the *R* increases, with the load ratio of *R* = 1 corresponding to the minimum *t_f_*. This finding indicates that bitumen exhibits a longer failure duration under unidirectional cyclic loading compared to creep testing. This phenomenon is attributed to the reduced accumulation of plastic strain during cyclic loading compared to static loading under the same maximum stress. Among all the tested *R* values, the bitumen exhibits the longest *t_f_* at *R* = 0.1 across all temperature conditions. Furthermore, the PG64-22 bitumen exhibits a longer *t_f_* than the PG58-28 bitumen under the same maximum stress, *R* value, and temperature, indicating its superior resistance to shear-induced plastic flow.

**Figure 5:**
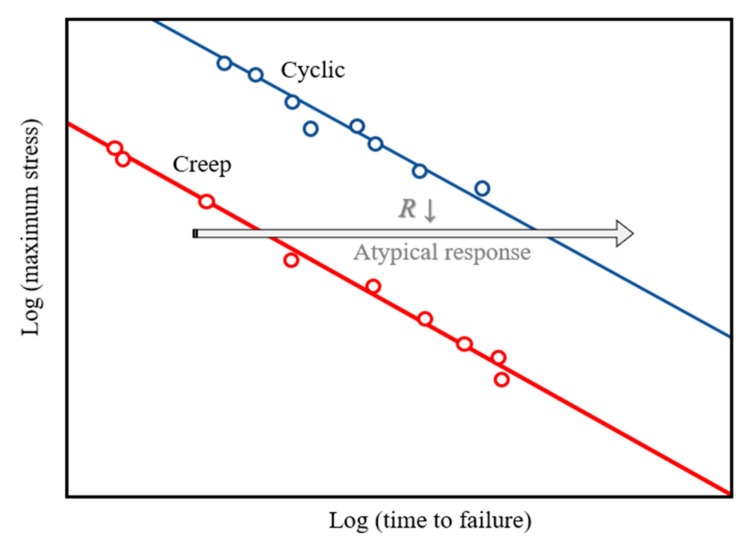
Schematic illustration of the “atypical response” [28].

Several researchers [28,29,30] have reported that the average stress level in unidirectional cyclic loading is lower than that in creep loading under the same maximum stress. Consequently, this results in reduced plastic accumulation and an extended material lifetime. This phenomenon, referred to as the “atypical response”, is illustrated in Figure 5. Kanters et al. [36] attributed the “atypical response” to a failure mechanism governed by the mean stress, referred to as plasticity-controlled failure. Moreover, several studies [31,32,36,37] have suggested that the *t_f_* is the most reliable characterization for evaluating a material’s failure in creep and cyclic tests. Therefore, this study investigates whether bitumen exhibits characteristics consistent with plasticity-controlled failure.

**Figure 6:**
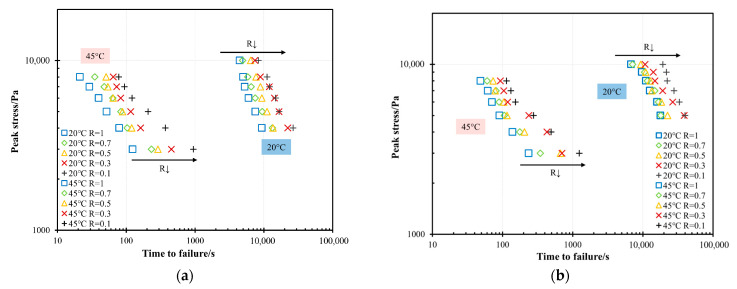
Time-to-failure of (**a**) PG 58-28 (**b**) PG 64-22 under different peak stresses and load ratios at 20 °C and 45 °C in the accelerated cyclic shear test.

**Figure 7:**
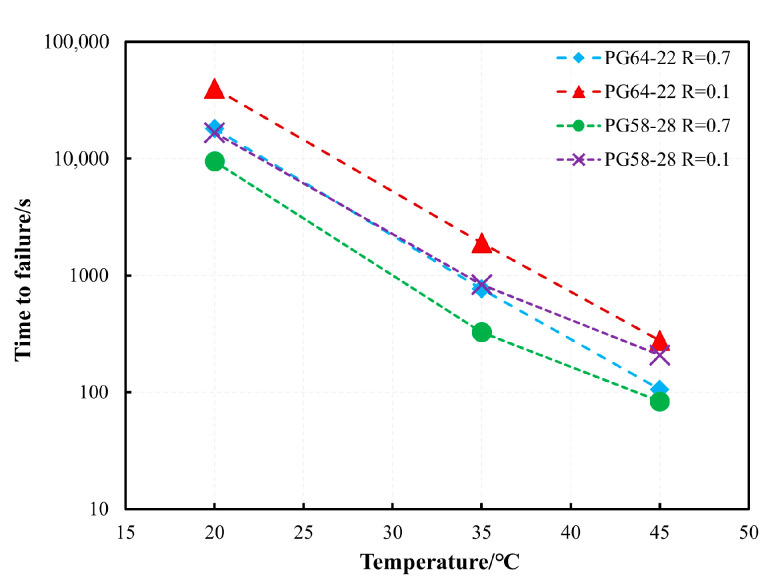
Time-to-failure at different temperatures of PG58-28 and PG64-22 bitumen in the accelerated cyclic shear test.

Figure 6 illustrates the relationship between the maximum stress and *t_f_* for PG58-28 and PG64-22 bitumen at 20 °C and 45 °C. As expected, an increase in maximum stress led to a gradual decrease in *t_f_* at the same temperature and *R* value. This phenomenon is attributed to elevated stress enhancing bitumen mobility, leading to a more rapid accumulation of plastic deformation over time and consequently a shorter *t_f_*. A key observation is that the evolutionary trend of the test data aligns entirely with the “atypical response” described above, indicating that the failure mechanism of bitumen under the accelerated cyclic shear loading is governed by plasticity-controlled failure. The influence of temperature on the *t_f_* of both bitumen types is also shown in Figure 6. With the increasing temperature, the plastic flow of bitumen intensifies, accelerating the accumulation of plastic deformation and leading to a shorter *t_f_*. Using a maximum stress of 5000 Pa as an example, Figure 7 illustrates the relationship between temperature and *t_f_* for PG58-28 and PG64-22 bitumen at *R* = 0.1 and *R* = 0.7. The results show that the *t_f_* at 20 °C is two orders of magnitude greater than at 45 °C, emphasizing the significant influence of temperature on bitumen’s fatigue failure.

### 3.2. Model Development

To develop a predictive model for the *t_f_* of bitumen in the accelerated cyclic shear test, a sigmoidal function, as presented in Equation (2), was employed to establish the relationship between the *R* and *t_f_* across a broad range of temperatures and stresses. The modelling process consists of two steps: a stress shift to a reference stress, followed by a temperature shift to a reference temperature. In the stress shift process, the relationship between *t_f_* and *R* for a given stress, represented in double logarithmic coordinates, can be horizontally shifted to a reference stress to construct a master curve. Similarly, in the temperature shift process, the *t_f_*-*R* relationship at different temperatures can be further shifted to generate a master curve at the reference temperature. However, when accounting for plasticity-controlled failure, these horizontal shifts deviate from the traditional time–temperature superposition principle applicable within the viscoelastic range [38,39].

(2) $$log\left|t_{f}\right|=a+\frac{b}{1+\frac{1}{{exp}^{d+elogR}}}$$

where *R* is the load ratio, *t_f_* is the time-to-failure, and *a*, *b*, *d*, and *e* are the regression coefficients.

### 3.2.1. Stress Shift

**Figure 8:**
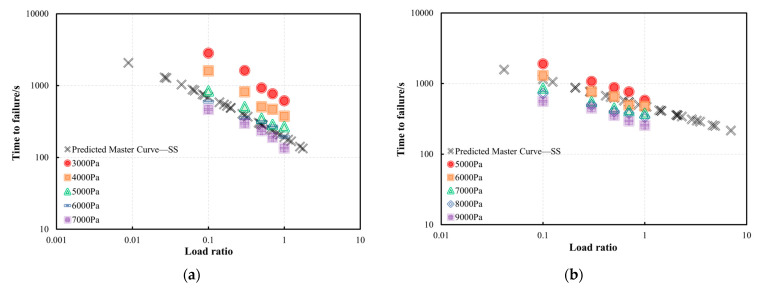
Master curves for (**a**) PG58-28 and (**b**) PG64-22 bitumen at 35 °C under reference stress of 6000 Pa.

**Figure 9:**
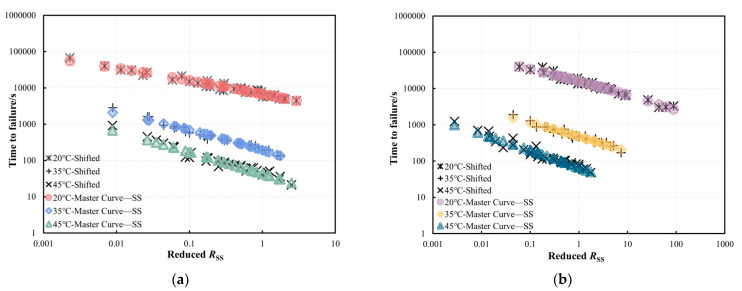
Master curves after the *SS* of (**a**) PG58-28 and (**b**) PG64-22 bitumen at 20 °C, 35 °C, and 45 °C under a reference stress of 6000 Pa.

**Figure 10:**
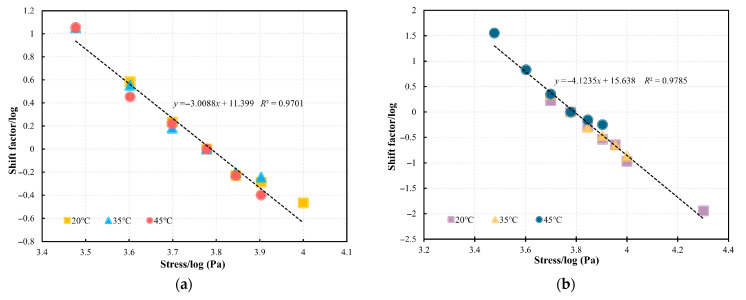
Shift factors of the *SS* of (**a**) PG58-28 and (**b**) PG64-22 bitumen at 20 °C, 35 °C, and 45 °C.

The master curves for PG58-28 and PG64-22 bitumen were initially developed through stress shift (*SS*) at 35 °C with 6000 Pa as the reference stress as an example, as shown in Figure 8. Once the master curve is established by the *SS*, the predicated *t_f_* under various stresses at 35 °C can be determined by using the developed failure prediction model that incorporates the *R* after shifting. For example, under low-stress conditions that are time-consuming, the *t_f_* can be estimated by performing cyclic tests at higher stresses with specific *R* values and applying appropriate shift factors. Similarly, the master curves for the two bitumen types at 20 °C and 45 °C referenced at a stress level of 6000 Pa can be constructed as shown in Figure 9. The obtained sigmoidal model parameters are listed in Table 3. The shift factors obtained from the *SS* for PG58-28 and PG64-22 bitumen are presented in Figure 10a,b, respectively. At 20 °C, 35 °C, and 45 °C, significant overlaps are observed in the relationships between the logarithmic shift factors and logarithmic stresses for both types of bitumen. This suggests that the relationship between shift factors and stresses established at a single temperature can be utilized to predict the *t_f_* at other temperatures. As a result, the influence of temperature can be neglected, allowing a unified linear relationship to be applied for fitting. In the subsequent sections, the fitted linear equations will be utilized to validate the failure prediction model.

### 3.2.2. Temperature Shift

**Figure 11:**
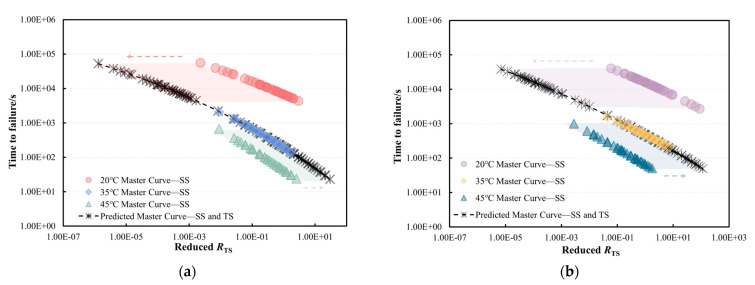
Master curves after the *SS* and *TS* of (**a**) PG58-28 and (**b**) PG64-22 bitumen under a reference stress of 6000 Pa.

**Figure 12:**
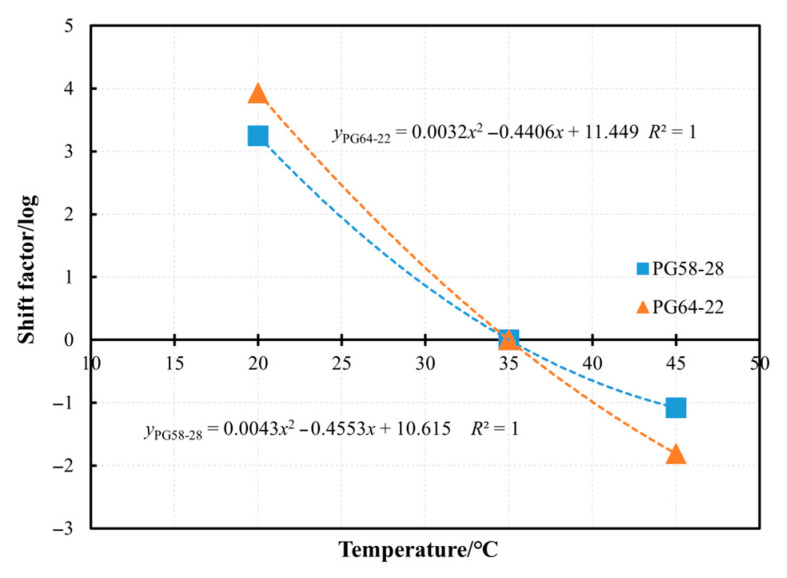
Shift factors of *TS* at different temperatures of PG58-28 and PG64-22 bitumen.

The master curves at 20 °C, 35 °C, and 45 °C, referenced at a stress level of 6000 Pa, can be horizontally shifted to construct a secondary master curve, referred to as temperature shift (*TS*). The fitting results are presented in Figure 11, while the fitted models for the two bitumen types are provided in Equations (3) and (4), respectively. Observation of these results suggests that the *t_f_* of bitumen at a given temperature can be predicted using data obtained at a different temperature. Specifically, cyclic test results from challenging conditions, such as extremely low temperatures, can be extrapolated from tests conducted at higher temperatures with reduced load ratios. Similarly, the *t_f_* at very high load ratios can be estimated by performing tests at lower temperatures. The relationships between shift factors and temperatures for PG58-28 and PG64-22 bitumen were fitted using quadratic polynomial functions, as shown in Figure 12. By integrating these fitting formulas with the master curve models, the *t_f_* can be predicted across various temperatures and stress levels.

(3) $$log\left|t_{f}\right|=-146.911+\frac{153.942}{1+\frac{1}{{exp}^{3.448-0.125logR_{TS}}}}$$

(4) $$log\left|t_{f}\right|=-1,573,957+\frac{206.797}{1+\frac{1}{{exp}^{3.482-0.0738logR_{TS}}}}$$

where *t_f_* is the time-to-failure and *R_TS_* is the reduced load ratio after *TS*.

### 3.3. Model Validation

**Figure 13:**
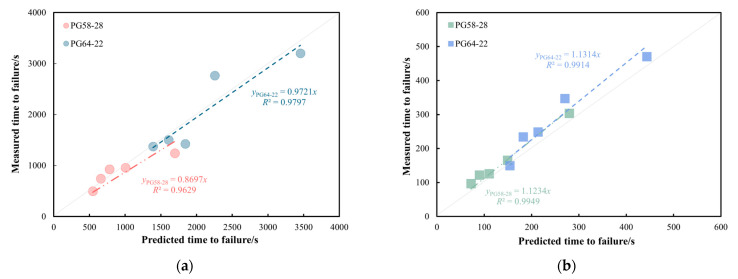
Comparison of the predicted and measured *t_f_* of PG58-28 and PG64-22 bitumen under 6000 Pa at (**a**) 30 °C and (**b**) 40 °C.

Based on the relationships shown in Figure 12, the corresponding shift factors and reduced reference load ratio (*R_TS_*) at a stress level of 6000 Pa can be determined, as presented in Table 4. By substituting the *R_TS_* into the fitted master curve models for PG58-28 and PG64-22 bitumen, represented by Equations (3) and (4), the *t_f_* at the validation temperatures of 30 °C and 40 °C can be predicted. Figure 13 compares the measured and predicted *t_f_* for the two bitumen types under a reference stress of 6000 Pa. The analysis demonstrates strong correlations between the predicted and measured values across various load ratios, confirming the accuracy of the failure prediction model in capturing the plasticity-controlled failure behaviour of bitumen under the new accelerated cyclic shear loading. At 30 °C, as shown in Figure 13a, the prediction model for the PG64-22 bitumen exhibits higher accuracy compared to that of the PG58-28 bitumen, achieving a correlation coefficient of 0.97. The models demonstrate excellent predictive accuracy of *t_f_* for both bitumen types at the verification temperature of 40 °C.

To predict the *t_f_* under various stresses at the verification temperatures of 30 °C and 40 °C, the relationship between shift factor and stress as shown in Figure 10 is used to determine the corresponding stress shift factors. The results presented in Table 5 show that the bitumen exhibits identical stress shift factors at same stress for the validation temperatures of 30 °C and 40 °C. The *R_TS_* that is calculated using Equation (5) is then incorporated into the master curve model developed at 35 °C, as defined by Equations (3) and (4), to predict the *t_f_* of PG58-28 and PG64-22 bitumen, respectively.

(5) $$R_{TS}=\frac{R}{\alpha_{SS}\alpha_{TS}}$$

where *R* is the load ratio—here *R*=0.1, 0.3, 0.5, 0.7, 1— *R_TS_* is the reduced load ratio after *TS*, *α_SS_* is the shift factor from *SS*, and *α_TS_* is the shift factor from *TS*.

**Figure 14:**
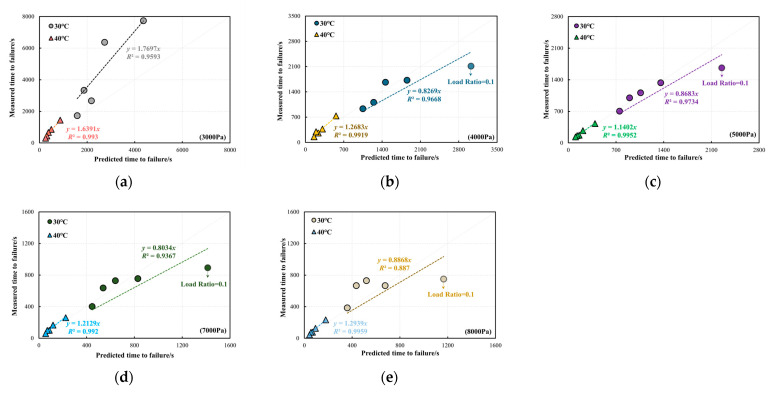
Comparison between the predicted and measured *t_f_* of PG58-28 bitumen at the validation temperatures of 30 °C and 40 °C: (**a**) 3000 Pa, (**b**) 4000 Pa, (**c**) 5000 Pa, (**d**) 7000 Pa, (**e**) 8000 Pa.

Figure 14 presents a comparison between the predicted and measured *t_f_* for the PG58-28 bitumen at the two validation temperatures. The results clearly indicate that at 30 °C, the bitumen exhibits a longer *t_f_* compared to 40 °C. Under a stress level of 3000 Pa, as shown in Figure 14a, the experimentally measured *t_f_* at both validation temperatures is higher than the values predicted by the model. This discrepancy is attributed to the slow plastic flow of bitumen under relatively low stresses, which allows self-healing to occur during the shear loading process. Under other stresses at 30 °C and 40 °C, the correlation between measured and predicted *t_f_* is strong, particularly at 40 °C. This indicates that the developed model reliably predicts the failure behaviour of bitumen. As observed in Figure 14, under the condition of 30 °C and *R* = 0.1, the predicted *t_f_* is generally higher than the measured values. This discrepancy arises as an *R* value of 0.1 represents the loading condition with the highest cyclic load amplitude among all cases examined in this study. Although the plastic accumulation of material under large-oscillation conditions is lower than under small-amplitude conditions, the fatigue behaviour induced by high-amplitude cyclic loading is particularly more pronounced. As a result, the measured *t_f_* for bitumen is shorter than the predicted *t_f_* under *R* = 0.1. However, at 40 °C the increased temperature enhances the plastic flow of bitumen, allowing it to absorb stress by plastic flow without accumulating significant fatigue damage. This effect is not fully captured in the predicted *t_f_*.

**Figure 15:**
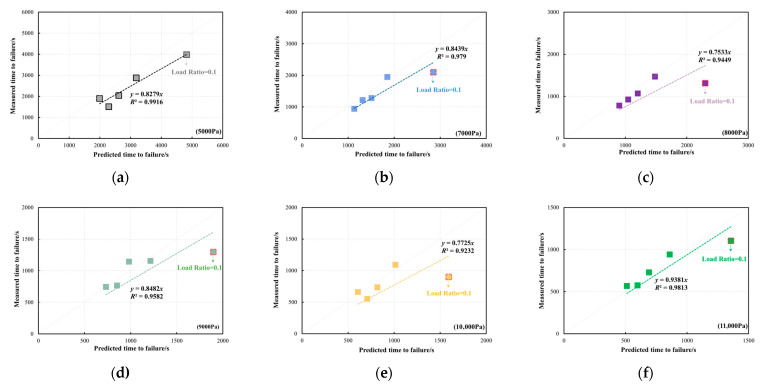
Comparison between the predicted and measured *t_f_* of PG64-22 bitumen at the validation temperature of 30 °C: (**a**) 5000 Pa, (**b**) 7000 Pa, (**c**) 8000 Pa, (**d**) 9000 Pa, (**e**) 10,000 Pa, (**f**) 11,000 Pa.

**Figure 16:**
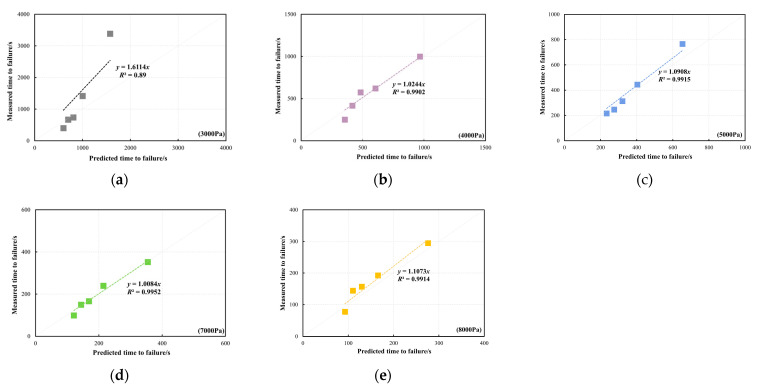
Comparison between the predicted and measured *t_f_* of PG64-22 bitumen at the validation temperature of 40 °C: (**a**) 3000 Pa, (**b**) 4000 Pa, (**c**) 5000 Pa, (**d**) 7000 Pa, (**e**) 8000 Pa.

Figure 15 presents a comparison between the predicted and measured *t_f_* of the PG64-22 bitumen at the validation temperature of 30 °C. The validation stresses range from 5000 Pa to 11,000 Pa with an increment of 1000 Pa. It can be noticed that the measured *t_f_* of the bitumen samples at *R* = 0.1 is lower than the predicted *t_f_* across different validated stresses. The comparisons between the predicted and measured *t_f_* of the PG64-22 bitumen at 40 °C are shown in Figure 16. A strong linear correlation is observed between the predicted *t_f_* from the developed master curve model and the measured *t_f_* across various validated stresses, except under the validated stress of 3000 Pa. Under a stress of 3000 Pa, the measured *t_f_* of the PG64-22 bitumen exceeded the predicted values, which is likely due to the self-healing behaviour of bitumen during the shear process under low stresses. However, this phenomenon was not observed at 30 °C due to the relatively higher initial stress. Similarly to the PG58-28 bitumen, a strong correlation between the predicted and measured *t_f_* is observed at the validation temperature of 40 °C, demonstrating the excellent prediction efficiency of the developed model.

## 4. Conclusions and Recommendations

### 4.1. Conclusions

This study proposed a novel accelerated cyclic shear test integrating both creep and unidirectional cyclic shear loading tests to investigate the fatigue behaviour and failure mechanism of bitumen. A new fatigue failure prediction model was developed to predict the fatigue life of bitumen. Based on the comprehensive experimental and modelling framework, the following conclusions can be drawn:

(1) The proposed novel accelerated cyclic shear test establishes a link between creep and cyclic loading modes by precisely controlling *R*. Experimental results demonstrated that lower load ratios (e.g., *R* = 0.1) significantly reduce plastic strain accumulation, resulting in notably extended *t_f_* compared to static loading (*R* = 1). This finding supports the existence of a plasticity-controlled fatigue failure mechanism, which is distinct from viscoelastic fatigue behaviour.
(2) The *t_f_* was validated as a robust and sensitive indicator for plasticity-induced fatigue failure. The observed “atypical response”—longer fatigue lifetimes at lower *R* despite identical peak stresses—is consistent with findings in thermoplastic and polymer fatigue research. This supports the assertion that mean stress significantly governs the evolution of failure in bitumen under these conditions.
(3) A master curve model based on a sigmoidal function incorporating stress and temperature shifts was developed and validated. Although the approach deviates from traditional time–temperature superposition principles used for the viscoelastic behaviour of bitumen, the temperature and stress shift techniques accurately predicted *t_f_* across a wide range of conditions. The model achieved high correlation coefficients (R^2^ > 0.95) at validation temperatures (30 °C and 40 °C), which confirms its predictive accuracy.
(4) The study employed two original bitumen types (PG58-28 and PG64-22), which were randomly sourced. Despite differences in their material properties, both showed similar fatigue trends and were well-described by the proposed model, with variations captured by model parameters. The results in this study provide evidence of the approach’s initial reproducibility and potential generalizability under the studied conditions. However, to further validate the robustness of the conclusions and ensure applicability across a broader range of materials, future work will extend the testing and modelling framework to include modified and aged bitumen.

### 4.2. Recommendations

Future studies should extend the current framework to include modified and aged bitumen, extended temperature ranges, and more complex loadings; incorporate microstructural material parameters into the master curve formulation; and validate across multiple laboratories and equipment platforms. Furthermore, coupling the macroscopic model with micromechanical or chemomechanical simulations could lead to a unified constitutive description of plasticity-induced fatigue in bitumen.

## Figures and Tables

**Table 1:** Properties of bitumen in this study.

Indicator	PG 58-28	PG 64-22
Flash Point (°C)	300	315
Viscosity at 135 °C (Pa·s)	0.42	0.47
RTFO Mass Loss (%)	0.36	0.40
G */*sinδ* (Original) (kPa)	1.23	1.35
G */*sinδ* (RTFO-aged) (kPa)	2.98	3.10

**Table 2:** Test matrix of PG58-28 (B1) and PG64-22 (B2) bitumen.

Maximum Load *τ*_max_ (Pa)	Load Ratio (*R*)	Temperature (°C)				
		20	30	35	40	45
2000	0.1, 0.3, 0.5, 0.7, 1				B1	B1, B2
3000		B1		B1	B1, B2	B1, B2
4000		B1	B1	B1	B1, B2	B1, B2
5000		B1, B2	B1, B2	B1, B2	B1, B2	B1, B2
6000		B1, B2	B1, B2	B1, B2	B1, B2	B1, B2
7000		B1, B2	B1, B2	B1, B2	B1, B2	B1, B2
8000		B1, B2	B1, B2	B1, B2	B1, B2	B1, B2
9000		B1, B2	B2	B2		
10,000		B1, B2	B2	B2		
11,000			B2			

**Table 3:** Sigmoidal model parameters of the *SS* of PG58-28 and PG64-22 bitumen.

Bitumen	Temperatures (°C)	Sigmoidal Model Parameters After *SS*			
		*a*	*b*	*d*	*e*
PG58-28	20	−57.582	65.280	2.765	−0.108
	35	−329.003	334.193	4.735	−0.216
	45	−165.391	176.014	2.922	−0.073
PG64-22	20	−49.260	56.536	2.855	−0.122
	35	−173.211	182.647	3.258	−0.061
	45	−173.521	185.299	2.869	−0.052

**Table 4:** Shift factors of PG58-28 and PG64-22 bitumen under reference stress of 6000 Pa.

Temperature (°C)	Temperature Shift Factors		Load Ratio *R*	Reduced *R_TS_*	
	PG58-28	PG64-22		PG58-28	PG64-22
30	6.699	12.912	0.1	0.0149	0.0077
			0.3	0.0448	0.0232
			0.5	0.0746	0.0387
			0.7	0.1045	0.05421
			1	0.1493	0.0774
40	0.192	0.0881	0.1	0.5212	1.1350
			0.3	1.5636	3.4050
			0.5	2.6060	5.6751
			0.7	3.6484	7.9451
			1	5.2120	11.3501

**Table 5:** Stress shift factors of PG58-28 and PG64-22 bitumen.

Verified Stress (Pa)	Stress Shift Factor (30 °C/40 °C)	
	PG58-28	PG64-22
3000	8.6504	19.9568
4000	3.6402	6.0940
5000	1.8601	2.4283
6000	1.0747	1.1449
7000	0.6759	0.6064
8000	0.4523	0.3496
9000	-	0.2151
10,000	-	0.1393
11,000	-	0.0940

## Data Availability

The original contributions presented in this study are included in the article. Further inquiries can be directed to the corresponding author.
